# Points

**Published:** 1898-06

**Authors:** 


### POINTS.

The United States army is a daily purchaser of horses in the great centres west. It is stated that each battery of artillery will require 100 horses, infantry regiments about 20 each, cavalry regiments about 1200 to 1300; each field-officer is entitled to two horses. Cavalry and artillery horses in the Chicago market are in strong demand, and contract prices range from $85 to $100 per head. Artillery horses must weigh from 1100 to 1250 pounds each, on the driving order finish, and of solid color. The government will buy only horses with full tails.

The National Horse-breeders', Dealers' and Exhibitors' Association, recently formed at Chicago, will urge government inspection of stallions used for public breeding purposes.

The *Breeders' Gazette* of May 18th strongly urges the giving of rank to the veterinarian in the army, and points forcibly to the changed conditions now than existed fifteen or twenty years ago, when there were but few educated veterinary surgeons.

Clay, Robinson & Co., of the Chicago stockyards, have offered one hundred dollars in gold for competition among the students of the Iowa Agricultural College in the judging of fat stock, including steers, wethers, and hogs.

Many of the reindeer imported for use in the Alaskan region are dying; lack of proper food and other conditions are operating causes.

At Chicago stockyards a charge of ten cents is levied on every horse entered, to cover the State veterinary inspection service.

The black hornfly has made its appearance in the central west, and cattle during the hot months are likely to make little flesh with this torment about.

A model of a Chicago stockyard sheep-dipping tank will be on exhibition at Omaha.

Secretary of Agriculture Wilson has called the attention of the Secretary of War to the importance of having good, competent veterinary forces with the United States cavalry. There are said to be few veterinarians attached to our army now who are graduates of regular veterinary schools. Secretary Wilson also calls attention to the fact that the United States Army is the only one in the civilized world in which veterinarians do not hold commissions. This is one reason, he thinks, why the most competent men in the profession have not sought service in the army.—*Breeders' Gazette*, May 25, 1898.

So-called "thumps" in pigs, due to excessive plethora of the neck and forequarters, arising from strong feeding and lack of exercise, a functional derangement of the circulation, is much benefited by changing the food to the less fattening kinds and the enforced exercise of those afflicted.

Last year Boston punished forty-eight offenders for selling impure milk.

The great Empire State (New York) boasts of having expended for the services of veterinarians and their expenses during the last eleven years, $6000.78. Surely her great livestock interests and public health must be of little moment if such a paltry sum was sufficient to guard these avenues of health and wealth.

The Northampton County (Pennsylvania) farmers have petitioned the State Livestock Sanitary Board for reimbursement of butchers who have purchased for slaughter, unknowingly, tuberculous carcasses, and thus remove one of the dangers of having this diseased meat thrown upon the market for consumption.

Chicago markets handled 98,000 horses in 1897; over 50,000 horses exported in 1897.

England, Ireland, Scotland, Belgium, Germany, France, Norway, Denmark, Sweden, Italy, Africa, Mexico, and South America are buyers of American-bred horses.

Spratts' Patent Company wish to announce to their customers that the recent advance in the price of wheat will not make any difference in price to their customers, owing to the fact that they were fortunate enough to secure long contracts for wheat; and also to answer the many inquiries as to the extent of the injuries of their Vice-President, Mr. G. G. Cleather, who was reported as being seriously injured, that the accounts were very much exaggerated, and that he is now quite well again.

The many friends of Mr. A. J. Cassatt will be rejoiced to learn that in the disastrous fire at Chesterbrook farm, the famous hackney stallion "Cadet," and the great stallion, "Bard," were gotten out in safety. The very valuable herd of Guernsey cattle were destroyed.

